# A study on the level of NLRP3 inflammasome in patients with premature rupture of membranes

**DOI:** 10.1002/iid3.1018

**Published:** 2023-09-20

**Authors:** Fen Xue, Yan Wang, Xue‐Qiang Tao, Wei Su

**Affiliations:** ^1^ Department of Obstetrics and Gynecology The Fourth Hospital of Baotou Baotou China; ^2^ Department of Science and Education The Fourth Hospital of Baotou Baotou China; ^3^ Department of Spinal Surgery The Fourth Hospital of Baotou Baotou China

**Keywords:** ASC, caspase‐1, IL‐1β, IL‐18, NLRP3, premature rupture of membranes

## Abstract

**Objective:**

In this study, we investigated the levels of interleukin‐1β (IL‐1β), IL‐18, and the NOD‐like receptor pyrin domain‐containing 3 (NLRP3) inflammasome in patients with premature rupture of membranes (PROMs).

**Methods:**

We selected 60 pregnant women at the Fourth Hospital of Baotou between January 2019 and July 2021. These women were divided into three distinct groups: the preterm PROM group with 20 cases, term PROM (TPROM) group with 20 cases, and a control group with 20 cases consisting of normal full‐term pregnancies without PROM. Peripheral blood was collected from all participants. Using enzyme‐linked immunosorbent assay, the levels of IL‐1 and IL‐18 in the plasma were assessed. Additionally, the proportions of NLRP3, apoptosis‐associated speck‐like protein (ASC), and caspase‐1‐positive macrophages were also evaluated.

**Results:**

The ratios of NLRP3, ASC, IL‐1β, and IL‐18 concentrations, along with the presence of caspase‐1‐positive macrophages, were notably greater in the PROM groups in comparison with the control group (*p* < .05). In the TPROM group and control group, the proportions of IL‐1β and IL‐18 levels were found to be lower than NLRP3, ASC, and caspase‐1‐positive macrophages levels (*p* < .05).

**Conclusion:**

The concentrations of IL‐1β and IL‐18, as well as the ratios of NLRP3, ASC, and caspase‐1‐positive macrophages, were elevated in patients with PROM compared to the control group. This suggests a potential correlation between the excessive activation of NLRP3 and the development of PROM.

## 1 INTRODUCTION

One of the most prevalent complications during pregnancy is the premature rupture of membranes (PROMs). Numerous factors that can lead to PROM, including infection, abnormalities in fetal membranes, and trauma.^1^, ^2^ In general, PROM is the result of a complex interplay of multiple elements,^3^ with reproductive tract infections being the primary contributor.^4^ Infection and PROM have a connected relationship: reproductive tract infections can trigger PROM, and conversely, PROM can pave the way for intrauterine and puerperal infections, as well as issues like umbilical cord prolapse, intrauterine fetal distress, and preterm birth. This, in turn, impacts the outcomes of pregnancy.^5^ PROM can be categorized into term PROM (TPROM) and preterm PROM (PPROM). Preterm labor brought about by PPROM constitutes about 35% of all preterm labor cases, and its occurrence is on the rise.^6^, ^7^ Dealing with PROM is one of the most challenging aspects in obstetrics, and the outlook for newborns is often unfavorable.^8^ Hence, investigating the underlying causes of PROM, initiating early prevention and intervention measures, and decreasing the incidence of PROM hold significant importance in improving maternal and delivery outcomes. The inflammatory response functions as a safeguarding immune reaction against external triggers. The notion of the inflammasome was initially introduced by Tschopp in 2002,^9^ representing a particular type of large molecular assembly initiated through caspase‐1 activation within the interior of immune cells. A notable example of this is the NOD‐like receptor pyrin domain‐containing 3 (NLRP3) inflammatory complex, which has been extensively investigated.^10^ This intricate protein composition involves NLRP3, apoptosis‐associated speck‐like protein (ASC), and caspase‐1 precursor elements. It is present in various types of immune cells within the bloodstream,^11^ serving as a link connecting inflammation and immunity.

The activation of this complex requires a linkage between the NLRP3 amino‐terminal thermoprotein domain and ASC. Following this connection, ASC recruits caspase‐1 precursors, forming an inflammatory complex that enhances the activation of caspase‐1. This activated caspase‐1 inhibits the maturation and release of interleukin‐1β (IL‐1β) and IL‐18 precursors, thereby impeding their progression.^12^, ^13^, ^14^ Additionally, it triggers a form of inflammatory cell death known as pyroptosis and releases more inflammatory molecules, thereby intensifying the inflammatory response even further.^15^ Overactivation of these inflammatory complexes may be linked to various inflammatory disorders that arise and progress.^16^ The occurrence of cell pyroptosis serves as a crucial mechanism the body employs to combat infections. A comprehensive understanding of the role cell death plays in infectious diseases may prove beneficial for managing such diseases clinically.

An animal model of lipopolysaccharide‐induced intra‐amniotic inflammation (IAI) demonstrated that there was priming of the NLRP3 inflammasome at the transcriptional and expression, indicated by enhanced messenger RNA and protein expression of inflammasome‐related genes; and using the specific NLRP3 inhibitor, MCC950 can inhibiting of the NLRP3 inflammasome reduced IAI‐induced preterm birth and neonatal mortality.^17^ Another study found that NLRP3 deficiency abrogated preterm birth and the resulting neonatal mortality induced by impeding the premature activation of the common pathway of labor as well as by dampening intra‐amniotic and fetal inflammation.^18^ However, the relationship between NLRP3 and PROM in the human still needs further clarification. Based on these researches, we speculate that the NLRP3 inflammatory complex heightens the activation of IL‐1β and IL‐18, ultimately leading to pyroptosis in fetal membrane cells and resulting in PROM. The presence of IL‐1β and IL‐18 in the bloodstream has been associated with the constitution of the NLRP3 inflammatory complex, underscoring the significance of investigating protein expression within this complex. Inflammatory complexes play a role in the progression and emergence of diverse infection‐related ailments, even though there is currently no documented information regarding the involvement of the NLRP3 inflammatory complex in PROM. In this study, we assessed the levels of IL‐1B and IL‐18 in blood plasma and analyzed the ratios of macrophages expressing NLRP3, ASC, and caspase‐1. Our aim was to better understand the NLRP3 inflammasome in patients with PROM. This investigation aimed to uncover the underlying causes and processes of PROM, ultimately laying the groundwork for dependable early‐stage clinical interventions.

## 2 DATA AND METHODS

### 2.1 Research participants

In this observational study, following the determination of the required sample size, we enrolled 60 pregnant women who received treatment at the Obstetrics and Gynecology Department of the Fourth Hospital of Baotou between January 2019 and July 2021. These women were divided into three distinct groups: the PPROM group with 20 cases, TPROM group with 20 cases, and a control group with 20 cases consisting of normal full‐term pregnancies without PROM.

The ethical committee of the hospital granted approval for the study, and written consent was obtained from all participants.

### 2.2 Inclusion criteria

The following were the inclusion criteria: (1) singleton pregnancy, cephalic presentation, no obvious cephalopelvic disproportion, no polyhydramnios, and no sex or trauma before membrane rupture; (2) absence of gestational diabetes, gestational hypertension, prior PPROM, or other complications during previous pregnancies; (3) no presence of infectious ailments like urinary tract or respiratory infections; (4) not having taken antibiotics or glucocorticoids within the preceding month; and (5) fulfilling the diagnostic criteria for both PPROM and TPROM.

The diagnostic criteria for PPROM and TPROM were in accordance with the ninth edition of *Obstetrics and Gynecology*, edited by Professor Xie Xing.^19^ These criteria consisted of the following: (1) following disinfection of the vagina and cervix, amniotic fluid became noticeable when the uterine fundus was touched by hand via the vaginal dilator, with direct visual observation; and (2) upon examination, the pH level of the vaginal discharge was alkaline, and the smear of fluid from the posterior vaginal fornix displayed fetal fat, fetal hair, fetal epithelial cells, or fern‐like crystals. PPROM is defined as the rupture of membranes (ROM) before labor before the 37th week of pregnancy, whereas TPROM is defined as ROM before labor between the 37th and 42nd weeks of pregnancy.

### 2.3 Methods

All women underwent a cesarean delivery and were instructed to fast overnight the previous day. Based on the inclusion criteria, blood samples were drawn from a peripheral vein on the morning before giving birth from all the study participants.

### 2.4 Determination of the expression level of IL‐1β and IL‐18 in serum

The participants were into three distinct groups. We employed a double‐antibody sandwich enzyme‐linked immunosorbent assay (ELISA) technique to detect the levels of IL‐1β and IL‐18 in serum. We subsequently compared the measured values between the different groups. The ELISA kits used in the analysis were procured from Hangzhou Lianke Biotechnology Co., Ltd. The ELISA procedure was meticulously conducted according to the provided reagent instructions.

For the testing process, both the samples and standard products were loaded into multiwell plates, with triplicate replications in separate wells. A volume of 3 mL of venous blood was collected from the upper limb of each of the 60 pregnant women. This blood was carefully collected into dry test tubes without any additives or anticoagulants to prevent disturbance, including vigorous shaking that could lead to hemolysis. The serum was subsequently extracted from these samples, which was centrifuged at 3000 rpm for 20 min as per a predetermined program. All collected samples were then stored at a temperature of −80°C for no more than 6 months.

Finally, the absorption value of the enzyme marker was read at 450 nm and the average value was calculated and a standard curve was established.

### 2.5 Detection of the proportion of NLRP3, ASC, and caspase‐1‐positive macrophages in the blood

We employed flow cytometry to determine the proportions of macrophages expressing NLRP3, ASC, and caspase‐1 in the three groups. We collected 3 ml of venous blood from the upper limb of each of the 60 pregnant women. To minimize vibrations, the venous blood was placed into a dry test tube without any additives or anticoagulants. The sample was then centrifuged at 3000 rpm for 20 min, leading to the separation of serum during this process. After removing the leukocytes, a double rinse with phosphate‐buffered saline was performed. Subsequently, the cells were fixed in 5% paraformaldehyde at room temperature for half an hour. Once fixed, the samples were treated with a blocking solution containing 10% goat serum, where the mouse antihuman primary antibody was allowed to incubate with the serum overnight at a temperature of 4°C. Following this, the samples were exposed to phycoerythrin‐Texas Red‐labeled rabbit antimouse secondary antibody at room temperature for 30 min. After this incubation, the cells underwent three washes with phosphate‐buffered saline containing Tween 20. The cells were then suspended in double distilled water and transferred to a flow tube. The macrophage subsets were analyzed using a BD LSRFortessa flow cytometer and the fluorescence intensity of NLRP3, ASC, and caspase‐1 on the cell surface was subsequently detected and assessed using FlowJo^TM^ 10 software, a flow‐cytometric analysis software.

### 2.6 Statistical analysis

The data analysis was performed using IBM SPSS Statistics for Windows, Version 22.0. Continuous variables are represented using the mean and SD ($\bar{x}\pm s$). The tests for normality and homogeneity of variance indicated that the data followed a normal distribution and had homogeneous variance, respectively. To compare groups, a one‐way analysis of variance test was employed. In cases where a statistically significant difference was observed, pairwise comparisons were conducted using the least significant difference method. Conversely, if the difference was not statistically significant, no pairwise comparison was undertaken. The comparison between the two groups was carried out using an independent sample *t* test. Data are presented as percentages (%) and the *χ* ^2^ test was utilized for the analysis. The proportion of NLRP3, ASC, and caspase‐1‐positive macrophages in the serum of the three groups were compared and analyzed. The results were deemed statistically significant if *p* < .05.

## 3 RESULTS

### 3.1 Comparison of general clinical data of research participants

There was no significant difference in the age and the frequency of previous childbirths among the group of 60 pregnant women (*p* > .05, as shown in Table 1). The gestational week, which represents the number of weeks at the time of sampling, was notably lower in the PPROM group compared with the other two groups and the difference was statistically significant (33.25 ± 0.556 weeks, *p* < .05, as indicated in Table 1).

**Table 1:** Comparison of general clinical data ($\bar{X}\pm S$).

Groups	PPROM group	PROM group	Normal group	*p*
*N*	20	20	20	
Age	28.80 ± 1.211	28.30 ± 0.877	28.95 ± 1.025	.917
Gestational week	33.25 ± 0.556	38.89 ± 0.296	39.18 ± 0.291	.000
Number of deliveries	1.20 ± 0.092	1.20 ± 0.086	1.25 ± 0.099	.908

Abbreviations: PPROM, preterm premature rupture of membrane; PROM, premature rupture of membrane.

### 3.2 Comparison of IL‐1β and IL‐18 levels between the PROM groups and the control group

The concentrations of IL‐1β (298.95 ± 4.72 pg/mL in the PPROM group and 149.43 ± 3.46 pg/mL in the PROM group) as well as IL‐18 (59.83 ± 0.46 pg/mL in the PPROM group and 46.76 ± 0.83 pg/mL in the PROM group) within the PPROM and control groups (IL‐1β: 85.31 ± 2.47 pg/mL and IL‐18: 38.72 ± 0.22 pg/mL) were notably lower compared with the TPROM group, and the difference was statistically significant (*p* < .05). The PPROM group exhibited elevated levels of IL‐1β and IL‐18 compared with the TPROM group, demonstrating a statistically significant difference (*p* < .05, as shown in Table 2).

**Table 2:** Determination of IL‐1β and IL‐18 in the three groups of pregnant women ($\bar{X}\pm S$) (pg/mL).

Groups	Normal group	PROM group	PPROM group
*N*	20	20	20
IL‐1β	85.31 ± 2.47	149.43 ± 3.46*	298.95 ± 4.72*, ** ^,^ **
IL‐18	38.72 ± 0.22	46.76 ± 0.83*	59.83 ± 0.46* ^,^ **

Abbreviations: IL‐1β, interleukin‐1β; IL‐18, interleukin‐18; PPROM, preterm premature rupture of membrane; PROM, premature rupture of membrane.

* *p* < .05 versus Normal group

** *p* < .05 versus PROM group.

### 3.3 Comparison of NLRP3, ASC, and caspase‐1‐positive macrophages between the PROM groups and the control group

The percentages of NLRP3‐positive macrophages (50.73 ± 0.51% in the PPROM group and 44.89 ± 0.41% in the PROM group), ASC‐positive macrophages (41.80 ± 0.68% in the PPROM group and 34.65 ± 0.34% in the PROM group), and caspase‐1‐positive macrophages (53.08 ± 0.51% in the PPROM group and 43.99 ± 0.43% in the PROM group) in the PPROM group and the TPROM group were significantly higher than in the control group (NLRP3: 39.30 ± 0.46%, ASC: 32.05 ± 0.26%, caspase‐1: 33.06 ± 0.51%), and the difference was statistically significant (*p* < .05). The proportion of NLRP3, ASC, and caspase‐1‐positive macrophages was higher in the PPROM group than in the TPROM group, and the difference was statistically significant (*p* < .05, as shown in Table 3).

**Table 3:** The proportion of NLRP3, ASC, caspase‐1‐positive macrophages in the three groups of pregnant women ($\bar{X}\pm S$) (%).

Groups	Normal group	PROM group	PPROM group
*N*	20	20	20
NLRP3	39.30 ± 0.46	44.89 ± 0.41*	50.73 ± 0.51* ^,^ **
ASC	32.05 ± 0.26	34.65 ± 0.34*	41.80 ± 0.68* ^,^ **
caspase‐1	33.06 ± 0.51	43.99 ± 0.43*	53.08 ± 0.51* ^,^ **

Abbreviations: ASC, apoptosis‐associated speck‐like protein; NLRP3, NOD‐like receptor pyrin domain‐containing 3; PPROM, preterm premature rupture of membrane; PROM, premature rupture of membrane.

* *p* < .05 versus Normal group

** *p* < .05 versus PROM group.

## 4 DISCUSSION

Based on the results of our study, we found that levels of IL‐1 and IL‐18 expression in the bloodstream were higher in women with PROM compared with pregnant women with no health issues. Based on this, it is proposed that patients with PROM may experience heightened activation of the NLRP3 complex. When comparing the three groups—women with TPROM, women with PPROM, and healthy pregnant women—it was observed that women with PPROM had notably more macrophages that tested positive for NLRP3, ASC, and caspase‐1. This difference was statistically significant, providing further evidence of a potential connection. The macrophage, a crucial cell of the innate immune system, is found in various body tissues. It possesses a potent ability to engulf and eliminate pathogens, including bacteria, through phagocytosis. At the same time, it acts as a presenter of antigens, triggering immune responses. Research underscores the vital role of macrophages in inflammation, where the intracellular inflammatory pathway serves as the first line of defense for the innate immune system. Pyroptosis, a form of cell death dependent on caspase‐1 produced by infected macrophages, leads to changes in membrane permeability and nuclear condensation.^19^, ^20^, ^21^ Currently, various mechanisms for PROM have been proposed. These encompass physical damage, infections in the reproductive tract, insufficient presence of trace elements, heightened expression of metalloproteinases, and the programmed cell death of fetal membrane cells, known as apoptosis. Among these, infection is a prominent factor contributing to PROM, as indicated by numerous studies. Pyroptosis, a specific type of programmed cell death, comes into play when the integrity of the cell membrane is compromised, leading to the release of inflammatory substances. This process is activated in response to either pathogenic infections or the stimulation of danger signals.^22^, ^23^ Pyroptosis hinges on the development of the NLRP3 inflammatory complex, which initiates an inflammatory cascade resulting in the secretion and release of IL‐1 and IL‐18.

The presence of PROM significantly heightens the risk of intrauterine infection and newborn mortality. This holds particularly true for PPROM, a complex challenge in obstetric diagnosis and care. Currently, researchers are focused on investigating its causes and underlying mechanisms to discover effective ways of predicting and intervening, aiming to enhance the management and treatment of patients with high‐risk of PRO. The results of this study illustrated notable differences in the levels of IL‐1 and IL‐18 expression, as well as the macrophage NLRP3 inflammatory complex, across the three groups. These levels were considerably elevated in PPROM cases compared with TPROM cases and healthy pregnant women. This suggests that an excessive activation of the inflammatory response may trigger a cascade of inflammation, ultimately leading to the pyroptosis of fetal membrane cells and thus PROM.

The discovery that patients afflicted with PROM exhibited higher quantities of IL‐1 and IL‐18, alongside increased proportions of NLRP3, ASC, and caspase‐1‐positive macrophages compared to those in the control group, implies a potential association between NLRP3 overactivation and the onset of PROM.

Our study has certain limitations, specifically, the small sample size, which needs to be addressed in forthcoming research. Furthermore, given the variation in gestational week at the time of sample collection between the groups, there is a need to include a study group in future investigations that matches both gestational age and body mass index.

Hence, there is a need for additional investigation into the molecular processes underlying cell pyroptosis. Prospective areas of study encompass the essential components within the cell pyroptosis pathway as well as pharmaceutical agents capable of controlling the initiation of inflammatory complexes. Such studies could provide a deeper understanding of how the body regulates its anti‐inflammatory immune capacity and the functions of immune cells like macrophages. These findings hold potential for utilization in anti‐infection treatments and may offer a novel approach to mitigating and managing PROM, particularly PPROM.

## AUTHOR CONTRIBUTIONS

*Conception and design of the research*: Fen Xue. *Acquisition of data*: Fen Xue, Xue‐Qiang Tao. *Analysis and interpretation of the data*: Fen Xue, Xue‐Qiang Tao. *Statistical analysis*: Xue‐Qiang Tao, Yan Wang. *Obtaining financing*: Yan Wang. *Writing of the manuscript*: Fen Xue, Wei Su. *Critical revision of the manuscript for intellectual content*: Wei Su. All authors read and approved the final draft.

## CONFLICT OF INTEREST STATEMENT

The author declare no conflict of interest.

## ETHICS STATEMENT

The study was conducted in accordance with the Declaration of Helsinki (as was revised in 2013). The study was approved by Ethics Committee of the Fourth Hospital of Baotou (No. BSYLL2022021). Written informed consent was obtained from all participants.

## Data Availability

The data that support the findings of this study are available from the corresponding author upon reasonable request.
